# Design of high-order antibiotic combinations against *M. tuberculosis* by ranking and exclusion

**DOI:** 10.1038/s41598-019-48410-y

**Published:** 2019-08-15

**Authors:** Kaan Yilancioglu, Murat Cokol

**Affiliations:** 1 Faculty of Engineering and Natural Sciences, Uskudar University, İstanbul, Turkey; 2 Laboratory of Systems Pharmacology, Harvard Medical School, Boston, Massachusetts USA; 3 Present Address: Axcella Health, Cambridge, Massachusetts USA

**Keywords:** High-throughput screening, Computational models, Combinatorial libraries, Pharmacodynamics, Cheminformatics

## Abstract

Combinations of more than two drugs are routinely used for the treatment of pathogens and tumors. High-order combinations may be chosen due to their non-overlapping resistance mechanisms or for favorable drug interactions. Synergistic/antagonistic interactions occur when the combination has a higher/lower effect than the sum of individual drug effects. The standard treatment of *Mycobacterium tuberculosis* (Mtb) is an additive cocktail of three drugs which have different targets. Herein, we experimentally measured all 190 pairwise interactions among 20 antibiotics against Mtb growth. We used the pairwise interaction data to rank all possible high-order combinations by strength of synergy/antagonism. We used drug interaction profile correlation as a proxy for drug similarity to establish exclusion criteria for ideal combination therapies. Using this ranking and exclusion design (R/ED) framework, we modeled ways to improve the standard 3-drug combination with the addition of new drugs. We applied this framework to find the best 4-drug combinations against drug-resistant Mtb by adding new exclusion criteria to R/ED. Finally, we modeled alternating 2-order combinations as a cycling treatment and found optimized regimens significantly reduced the overall effective dose. R/ED provides an adaptable framework for the design of high-order drug combinations against any pathogen or tumor.

## Introduction

Combinations of three or more drugs are frequently used in the treatment of pathogens and tumors. Such high-order combinations are generally constructed by combining drugs that are effective and with non-overlapping resistance mechanisms^1^. For example, a combination of ethambutol, isoniazid and rifampicin (EIR), drugs with distinct mechanisms of action, is the standard treatment against *M. tuberculosis* (Mtb)^2^. Similarly, the standard treatment against Diffuse Large B-Cell Lymphoma is a combination of five drugs (R-CHOP) with different targets^3^. A treatment with many distinct drugs impose multiple bottlenecks for resistance evolution in heterogeneous cell populations^4,5^. This 50-year-old hypothesis has been the main design element for high-order drug combinations and has been recently supported by empirical evidence^1^.

Combinations of drugs may interact, that is, the combination’s effect may be significantly different than the sum of individual effects^6^. If the combination’s effect is more, equal or less than the sum of individual effects, the combination has synergistic, additive or antagonistic interaction, respectively^7,8^. Synergistic combinations allow for dose-reduction thereby alleviating high-toxicity, or dose-escalation for restricting resistance evolution^9,10^. Despite the high potential of high-order synergistic combinations for treatment improvement, a very large portion of studies focus on combinations of only two drugs. The number of possible ways to combine a set of n drugs is 2^n^, there are only (n choose 2) combinations with two drugs. For example, there are 1,048,576 mathematically possible combinations among 20 drugs while there are only 190 possible pairwise combinations. A number of comprehensive pairwise drug interaction screens included all pairwise interactions around 20 drugs^11–14^. These screens showed that synergy is rare, while antagonism was the most common interaction type. Cheminformatics and bioinformatics analyses of drug interactions and cellular functions have produced a compendium of methods to predict if a drug pair will be synergistic^15–20^.

Recently developed methods to measure high-order drug interactions showed that the standard treatments for Mtb and B cell lymphoma (EIR and R-CHOP) were not synergistic^1,21^. These methods also allowed testing of very large numbers of high-order interactions; for example a recent study has measured 251 two-drug combinations, 1,512 three-drug combinations, 5,670 four-drug combinations, and 13,608 five-drug combinations^22^. However, the exponential increase in possible combinations precludes comprehensive experimental screens of high-order synergies even for a small number of drugs^23^. Several computational studies reported reliable estimations for high-order interactions using only pairwise interaction data^21,24–26^. A recent study successfully applied this method to drug interactions in Mtb and to combinations of up to 10 drugs^27^. Therefore, testing all 190 pairwise interactions among 20 drugs is adequate to make estimations for the remaining 1,048,531 combinations.

Mtb claims more than 1 million lives every year. Mtb strains resistant to standard treatment pose a growing threat to global health^28^. Here, we experimentally measured all 190 pairwise interactions among 20 antibiotics in Mtb. We used this information to rank all high-order combinations among these antibiotics by their synergy. In addition, we introduced criteria for the exclusion of combinations with overlapping mechanisms from treatment options. Using this framework, we explored how to improve the current Mtb treatment by using high-order antibiotic combinations. Our ranking/exclusion design (R/ED) framework proposes a pipeline for nominating best high-order combinations and provides several test cases for treatment of drug-resistant Mtb.

## Results

### Measurement of all pairwise static interactions among 20 antibiotics in *M. tuberculosis*

**Figure 1:**
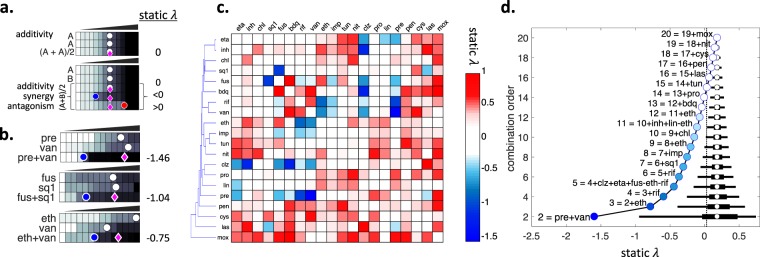
Static interactions among 20 antibiotics against *M. tuberculosis*. (**a**) Experimental setup for pairwise drug interaction measurements. *M. tuberculosis* cells are grown on increasing concentrations of single drugs and drug combinations. In each row, a circle marks the dose where a given inhibition level is observed (e.g. IC70 = 70% Inhibitory Concentration). The magenta diamonds indicate the well that is expected to achieve IC70 given the single drug IC70s. We scored each pairwise combination by *static λ* = log2(obs/exp). (top) By definition, a drug’s combination with itself is additive. In this case, the observed and expected IC70s will be identical and *static λ* = 0. (bottom) When two different drugs are combined, if observed IC70 is less or more than expected IC70, *static λ* scores are negative or positive, indicating synergy or antagonism. (**b**) Three examples of synergy observed among 190 tested pairwise interactions. The observed IC70 in the combinations pre + van, fus + sq1 and eth + van (blue circles) is strikingly less than the expected IC70 given single drug IC70s (magenta diamonds), resulting in negative *static λ* scores. (**c**) Hierarchical clustering of all interaction scores among 20 antibiotics are shown as a heatmap. Blue, white or red indicate synergy, additivity or antagonism, respectively. Among 190 pairs tested, 11 pairs had *static λ* < −0.5. (**d)** The effect of combination order (number of drugs in a combination) on the distribution of static interaction scores (*static λ*). Black horizontal line indicates the 95% and 50% percentiles for the interaction scores, and white circle shows the mean of all interaction scores. The best combination in each combination order is indicated with a circle on the left, named in reference to the best combination in the previous order. Vertical dashed line marks the *static λ* value estimated for the 3-order combination eth + inh + rif, the current Mtb treatment.

We conducted a large-scale experimental screen to measure all 190 pairwise interactions among 20 antibiotics against Mtb growth *in vitro* (Table 1). Our list of antibiotics included 3 drugs commonly used to treat Mtb (ethambutol, isoniazid, rifampicin) and 11 drugs that are considered or developed as treatments against Mtb. To achieve a broad sampling of drug and drug interaction space, we included six well-studied antibiotics (chloramphenicol, fusidic acid, nitrofurantoin, pentamidine, tunicamycin, vancomycin) which are not usually used for Mtb treatment. We collected optical density measurements to track the growth of Mtb cells after 5 days under 20 single drugs and 190 two-drug combinations. Using this data, we calculated a score (*static λ*) for each drug pair (Methods, Fig. 1a) which quantifies synergy-antagonism for the bacteriostatic effect. *Static λ* is 0 for additive drug pairs. Negative or positive values indicate synergy or antagonism, respectively. In line with previous studies, we define drug combinations with static interaction scores less than −0.5 as synergistic combinations^11,21^.

**Table 1:** Names, abbreviations, PubChemID and top doses for 20 compounds used in this study.

name	abbreviation	pubchemID	top dose (μg/ml)
bedaquline	bdq	5388906	0.6
chloramphenicol	chl	5959	12
clofazimine	clz	2794	2.8
cycloserine	cys	6234	5
ethambutol	eth	14052	1.5
ethionamide	eta	2761171	3
fusidic acid	fus	3000226	24
imipenem	imp	104838	20
isoniazid	inh	3767	0.18
lassomycin	las	132528125	0.54
linezolid	lin	441401	1.2
moxifloxacin	mox	152946	0.35
nitrofurantoin	nit	6604200	4
pentamidine	pen	4735	4.5
pretomanid	pre	456199	0.8
prothionamide	pro	666418	0.7
rifampicin	rif	5381226	0.06
SQ109	sq1	5274428	1.8
tunicamycin	tun	11104835	3
vancomycin	van	14969	5.2

In accordance with numerous recent drug interaction screens in various species, we found synergy was rare and antagonism was common. In total, we identified only 11 synergistic combinations among 190 tested pairs. Three of these synergies have been previously reported and the remaining eight synergies are novel^21^. Three static synergies identified (pre + van, fus + sq1 and eth + van) in this study are shown in Fig. 1b. The first two examples offer >50% reduction of total drug dose to observe the same inhibitory effect. Figure 1c shows all 190 pairwise static interaction scores among 20 antibiotics. 10 drugs in our screen never exhibited drug synergy. 5 of 11 synergies included clz, making this drug a promiscuous synergizer^12^. Among all the pairwise combinations tested in our screen, pre + van was the best pairwise combination for bacteriostatic effect against Mtb.

### Estimation of all high-order static interactions among 20 antibiotics in *M. tuberculosis*

We define combination order as the number of drugs in a combination. For example, standard treatment of Mtb is a 3-order combination (eth + inh + rif). Following the methodology used in^21^, we used the arithmetic mean of *static λ* of all 2-order combinations among constituents of a high-order combination as an estimation for the high-order combination’s *static λ*. For example, *static λ for* eth + inh + rif (EIR) is 0.08. Of three 2-order combinations possible among these three drugs, eth + rif is synergistic and eth + inh is antagonistic, making this 3-order combination additive.

Using this operation, we estimated *static λ* scores for each of the 1 million possible combinations, for all order combinations from 3 to 20. Figure 1d shows the 2.5–97.5 and 25–50 percentiles of *static λ* scores for each combination order from 2 to 20. As expected, the mean *static λ* for all combination orders is equal and slightly antagonistic. The distribution has a smaller range with increasing combination order, because interactions of opposite signs in each high-order combination tend to cancel each other, as in EIR. As a corollary, the higher the combination order, the higher the best achievable *static λ*; indicating that the strength of the best synergy decreases with increasing combination order. On the left of each distribution, the combination with the best score is shown. pre + van is the best 2-order combination, as stated above. The best 3-order combination has eth in addition to pre + van. The best 4-order combination has rif in addition to eth + pre + van. However, the best 5-way combination (clz + eta + fus + pre + van) has neither eth or rif, which were included in the 3 and 4-order best combinations. While the best combination has progressively worse *static λ* score with increasing combination order, we found synergistic 3 and 4-order combinations (*static λ* < −0.5). In addition, we were able to find combinations of up to 14 drugs that have stronger synergy than EIR.

pre + van is the best 2-order combination with a *static λ* of −1.5 and eth + pre + van is the best 3-order combination with a *static λ* of −0.8. While it is correct to say that pre + van has a stronger synergy than eth + pre + van, it is incorrect to say that pre + van is preferable to eth + pre + van for treatment of Mtb. The latter combination has three drugs and provides more bottlenecks for resistance evolution than the former combination, which has two drugs. Therefore, a ranking of combinations for best therapeutic value is possible only within each combination order. Increasing the number of component drugs increases the number of resistance bottlenecks, with the tradeoff of decreased level of synergy for most combinations.

### Measurement and estimation of lytic interactions among 20 antibiotics in *M. tuberculosis*

**Figure 2:**
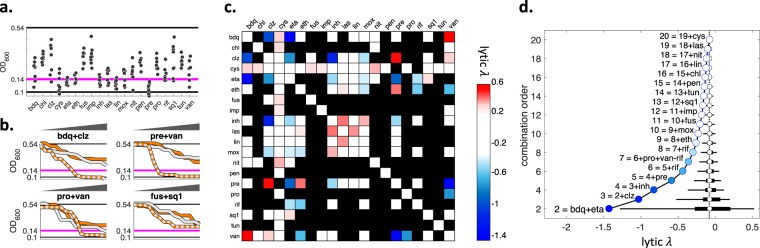
Lytic interactions among 20 antibiotics against *M. tuberculosis*. (**a**) OD600 readings at the top concentrations for 20 individual drugs are shown. The cells have an OD600 of 0.14 in Day 0 (magenta line) and untreated cells grow to an OD600 of 0.54 by day 5 (top black line). At the highest concentration of some drugs, the OD600 drops below 0.14 and can be as low as 0.1, OD of media with no cells (bottom black line). For example, cys, eta, inh, lin and pre each reduce the cell population below the magenta line. We used OD = 0.14 as initial cell number, OD = 0.1 as no cells, and OD = 0.12 as the phenotype where 50% of the cells are lysed. The concentration of a drug that results in 50% lysis is defined as LC50 (50% lytic concentration). (**b**) Pairwise lytic interaction examples. We used the same methodology to measure *static λ* scores to measure *lytic λ* scores, after two modifications: we used LC50 for score calculation; when 50% lysis was not observed in neither singles nor the combination, we were unable to calculate a *lytic λ* score. In each subplot, single drugs are shown with white and orange lines, combination is shown with a two-color line. bdq + clz and pre + van was previously shown to have static synergy. These pairs also have lytic synergy since each pair has a lytic effect while neither of the singles do. Static and lytic synergy do not always co-occur. For example, pro + van does not have static synergy, however it has lytic synergy. fus + sq1 has static synergy but has no lytic synergy. (**c**) Heatmap of lytic interaction scores among 20 antibiotics. Blue, white or red indicate synergy, additivity or antagonism, respectively. Black squares are pairs for which *lytic λ* was not calculable. Among 190 pairs considered, only 9 were found to have lytic synergy (*lytic λ* < −0.5). (**d**) The effect of combination order on the distribution of lytic interaction scores. Description and interpretation are identical to Fig. 1d.

To provide insight into the effect of drug combinations on cell lysis, we next analyzed the Mtb growth data collected in our screen for the lysis phenotype and calculated *lytic λ* scores that indicate lytic synergy or antagonism. We observed that for some drugs and combinations, the OD readings at Day 5 decrease compared to Day 0. Cycloserine, isoniazid and pretomanid Day 5 measurements are smaller than the beginning of the experiment (Fig. 2a). The optical density never goes below 0.1, which corresponds to the cell media’s absorbance. We therefore defined the decrease of optical density below the initial inoculation as lytic effect.

The lysis phenotype is evaluated with the same methodology as the interaction for growth inhibition (static *λ*) (Methods); with negative or positive values corresponding to synergy or antagonism, respectively. When lysis is not observed under either individual drugs or the combination, the *lytic λ* score cannot be calculated. *lytic λ* < −0.5 is defined as lytic synergy. Lytic interaction scores are inferred from a very low dynamic range, where no lysis is 0.14 and full lysis is 0.1. To establish that we can get reproducible lytic interaction scores, we compared the interaction scores among replicates. Scores from two replicates had a significant correlation (Spearman r = 0.85, p = 1.1 × 10^−8^), indicating that optical density measurements at such low resolution provide accurate estimates for the lysis phenotype. The combinations bdq + clz and pre + van exhibit lytic synergy, despite none of the individual drugs having lytic effect even at their highest doses (Fig. 2b, top row). These two drug pairs also have static synergy, so they are combinations with both static and lytic synergy. Lytic and static synergy do not always co-occur (Fig. 2b, bottom row): For example, pro + van has lytic synergy but does not have static synergy. Conversely, fus + sq1 has static synergy but does not have lytic synergy. All pairs for which *lytic λ* scores could be calculated using our data set are presented as a heatmap in Fig. 2c. In total, we produced *lytic λ* scores for 72 of the tested 190 pairs. Similar to static synergy, lytic synergy was also rare, with only 9 pairs having *lytic λ* < −*0.5*.

We produced estimates for the *lytic λ* scores of high-order combinations by using the same methodology we used for calculating high-order *static λ* estimates (Methods). Figure 2d shows the distribution of *lytic λ* scores in each combination order. Our analysis identified lytic synergy (*lytic λ* < −0.5) in combination orders up to five. In parallel with our observations for static synergy in Fig. 1d, the range of *lytic λ* distributions get smaller and best lytic synergy gets weaker by increasing combination order. However, the best synergy identified for different combination orders can be very different for static and lytic synergy. For example, the best 4-order static synergy (eth + pre + rif + van) and the best 4-order lytic synergy (bdq + clz + eta + inh) are composed of entirely different antibiotics.

A recent publication has reported experimental data for high-order drug interactions among up to five antibiotics in Mtb, using the diagonal method employed in our screen^27^. We computed static and lytic *λ* scores for these combinations. R/ED predictions, which only used the mean of all pairwise interaction scores among constituent drugs in a combination, significantly correlated with the obtained experimental scores (static synergy, r = 0.78, p = 1.7 × 10^−3^; lytic synergy, r = 0.89, p = 1.6 × 10^−5^). Therefore, this experimental data provides support for our model and its predictions.

### Drug interaction profile correlation as a proxy for drug similarity

**Figure 3:**
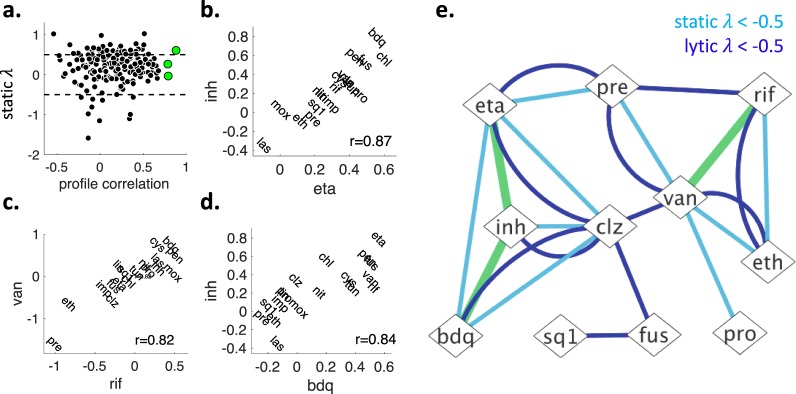
Finding drug pairs with similar drug interaction profiles and snapshot summary of pairwise static-lytic synergies and profile-similarity. **(a**) X-axis shows the *static λ* score profile correlations for drug pairs. Profile correlation is high for two drugs with similar drug interaction profiles. Y-axis shows the *static λ* for two drugs, which is negative, zero, or positive for synergistic, additive and antagonistic drug pairs, respectively. Each black circle indicates one pairwise drug combination. Three red circles show three drug pairs whose profiles were significantly correlated after Bonferroni correction (p < 2.6 × 10^−4^). Profile similarity of these pairs are visualized in (**b–d**), with the corresponding Spearman correlation scores. We define these pairs as “similar drugs”. (**e**) After the analysis of 190 pairs involving 20 drugs, we found 11 pairs with static synergy (*static λ* < −0.5, light blue edges), 9 pairs with lytic synergy (*lytic λ* < −0.5, dark blue edges) and three similar drug pairs (green edges).

Drug pairs with similar known mechanism of action have been repeatedly shown to have similar drug interactions^11,15,29^. A corollary of this finding is that drugs that have similar drug interactions will have similar mechanisms of action^30–32^. This corollary groups similar drugs together in an unsupervised fashion and can be used to assign mechanism of action to unstudied drugs. We used drug interaction similarity as exclusion criterion for high-order drug combination optimization to minimize the probability of shared mechanism of drug resistance. To the best of our knowledge, drug interaction similarity has not been used as a proxy for shared drug resistance mechanisms in other systems. As a measure of drug interaction similarity, we calculated the correlation between the *static λ* profiles of two drugs (profile correlation) for each drug pair in our analysis. Figure 3a shows that there is no clear relationship between profile correlation and the *static λ* scores of drug pairs. Profile correlation ranged from −0.5 to almost 0.9, and three drug pairs had significantly similar interactions after Bonferroni correction (p < 2.7 × 10^−4^): eta + inh, rif + van, bdq + inh. The relationship between the profiles of these pairs is shown in Fig. 3b–d. In this study, we will define these three drug pairs with significant profile correlation as similar drugs.

### Ranking and exclusion for selecting best drug combinations

Figure 3e shows a network representation of all static and lytic 2-order synergistic combinations. As noted earlier, a very small fraction of all 190 tested pairs showed *static or lytic synergy*. There is an overall regression toward additivity, therefore an even smaller fraction of all high-order combinations is synergistic. However, since the number of possible combinations in high-orders is very large, there are many combinations with high-order synergy. For example, 16 and 24 combinations show static and lytic synergy, respectively, among 1140 possible 3-order combinations. As shown for 4-way best combination with examples, static and lytic synergy can be very different. Above, we have produced *static λ* and *lytic λ* scores for all 2^20^ combinations among 20 antibiotics (Additional File 1). In order to optimize for both static and lytic synergy, we ranked all combinations by their *static λ* or *lytic λ*. Then we summed the ranks of combinations in static or lytic ladders and defined this as a meta-rank. The combinations with a lower meta-rank has better static and lytic synergies. At the end of this ranking, we report the best combination from each combination order.

Figure 3e also shows the drug pairs that were similar. We use this information to generate criteria for the exclusion of some combinations from further consideration. Imagine that a and b are similar drugs. If a + b + c and a + c + d have equal meta-ranks, then we regard a + b + c inferior to a + c + d, because the former would hit only two mechanisms of action while the latter would hit three. In another example, if a + c and a + b + c have equal meta-ranks, both these combinations would be hitting two mechanisms of action. Therefore, we regard a + b + c inferior to a + c, since it brings in a new drug with no added benefit of reducing total dose or constricting resistance evolution. As a corollary, all combinations that include similar drugs has a meta-rank inferior to at least one combination of one lower-order. In this light, we exclude all such combinations from consideration. This base exclusion criterion eliminates more than 60% of all possible combinations among 20 drugs, leaving 393,195 combinations to choose from. Additional exclusion criteria can be used for specific optimization problems. Below, we will find best high-order combinations in several test cases using ranking/exclusion design (R/ED).

### Improving the canonical *M. tuberculosis* treatment by adding new drugs

As noted above, the standard treatment against Mtb is a combination of three drugs: eth + inh + rif (EIR). Here, we will use R/ED to find which single drug would the best addition to EIR. This is equivalent to finding the best 4-way combination that includes EIR. To find this combination, we use two additional exclusion criteria: (i) EIR is a subset of the combination, and (ii) combination order = 4.

**Figure 4:**
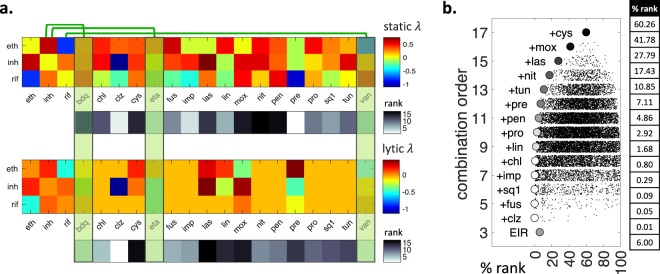
Improving the current *M. tuberculosis* treatment by adding new drugs. (**a**) At the top, static interactions of eth, inh and rif (EIR) with the other 17 drugs are shown. Green vertical rectangles indicate drugs that are similar with a member of EIR. inh is similar to bdq and eta, rif is similar to van. Therefore, bdq, eta and van are excluded from further consideration. The estimated static interaction score for each 4-order combination is ranked and shown in black-white. Pre and clz rank high among 4-order combinations that include EIR. At the bottom, lytic interactions for EIR with other 17 drugs are shown. Unknown interactions are shown in orange. The estimated lytic interaction score for each 4-order combination is ranked and shown in black-white. Clz ranks high in lytic synergy, however pre ranks low due to its antagonistic lytic interaction with eth. Therefore, clz emerges as the top ranked compound to complement EIR. (**b**) Relationship between combination order and rank% among all combinations including EIR. %rank for all combinations in each combination order is shown as a scatter plot jittered in y axis. The combination with best %rank in each order is indicated with a circle, with its color corresponding to its %rank among all possible combinations. EIR is the only 3-order combination and ranks at 6%. Addition of clz greatly improves the static and lytic synergy ranks of EIR, and 4-order combination EIR + clz is ranked at 0.01%. A 6-order combination involving EIR + clz + fus + sq1 ranks in the first 0.1%.

Since there are 20 drugs in our analysis, there are 17 drugs that can be added to EIR to make a 4-order combination. However, due to base exclusion criterion, three of these combinations (EIR + eta, EIR + bdq, EIR + van) are disregarded from further consideration, leaving only 14 combinations to choose from. In Fig. 4a top, *static λ* scores of EIR with all other drugs is shown. There are only two synergies among candidate drugs and a member of EIR: clz + inh and pre + rif. las and mox are both antagonistic with inh. Below the *static λ* panel, we show a heatmap corresponding to the rank of the 17 considered 4-order combinations. For example, EIR + pre and EIR + clz have low ranks, since they both have synergy with a member of EIR. However, EIR + pre is ranked best as clz is slightly antagonistic with eth. Similarly, EIR + las and EIR + mox is ranked high since las and mox show overall antagonism with EIR members. In Fig. 4a bottom, the same analysis is repeated with *lytic λ* scores. The only lytic synergy in this subset is between clz and inh, which makes EIR + clz the best 4-way combination for lytic synergy. EIR + pre, which is the best pair for static synergy, has a very high lytic rank since (i) pre + rif, which has static synergy, does not have lytic synergy and (ii) eth + pre, which is static additive, is lytic antagonistic. Next, we calculate the meta-rank for each EIR + x combination by summing the static and lytic ranks. EIR + pre’s static and lytic ranks are 1 and 10, respectively, giving EIR + pre a meta-rank of 11. EIR + clz’s static and lytic ranks are 1 and 2, respectively, giving this combination a meta-rank of 3. As this is the lowest meta-rank among 17 considered 4-order combinations, we declare clz the best drug to add to the canonical Mtb treatment.

We next expanded our search by removing one of the additional exclusion criteria, that the combination must have four drugs. When all combination orders are considered, there are 131,072 combinations that include EIR. We found the combination with the best meta-rank in each order using R/ED. Figure 4b shows the distribution of combination meta-ranks in each combination order. EIR is the only and best 3-order combination considered in this case. EIR + clz was previously established to be the best 4-order combination. The best combination in each next order is achieved by the addition of a single drug to the best combination in the previous order. For example, best 5, 6, and 7-order combinations are achieved by adding fus, fus + sq1, and fus + sq1 + imp, respectively, on EIR + clz. We transform meta-score ranks to %rank for easy interpretation. On the right of Fig. 4b, the %rank of the best combination in each order is given. The %rank of EIR is 6, which means EIR is only at the top 6% among all combinations for static and lytic synergy. EIR + clz is at top 0.01 percentile, and is the best combination among all combination orders. For *static λ* and *lytic λ* we have previously observed that with increasing combination order, there is a penalty for best synergy and there is a reward with resistance restriction. For this reason, we were unable to compare the best combinations from different orders. In this specific case, since the 4-order best combination has better synergy than the 3-order best combination, there is a reward in both synergy and resistance restriction. As a result, we conclude that EIR + clz is a better combination than EIR. However, starting with the 5-order combination EIR + clz + fus, %rank of the best combination declines and we can no longer compare best combinations from different orders.

### Exploring best 4-order combinations against drug-resistant *M. tuberculosis*

**Figure 5:**
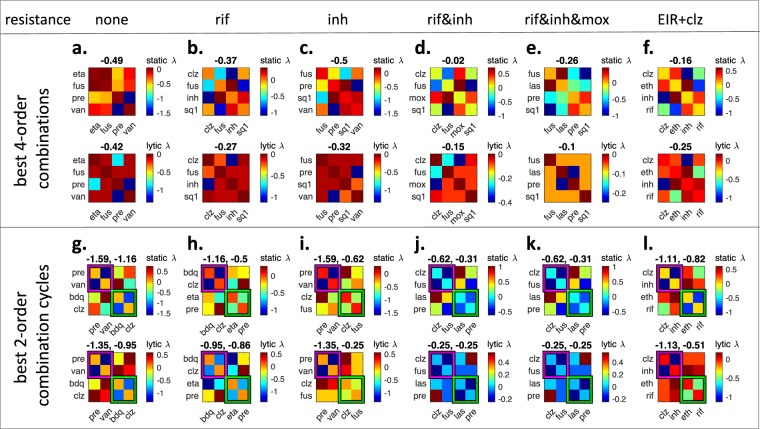
Exploring best 4-order combinations and 2-order drug cycling options against drug-resistant *M. tuberculosis*. In each panel, all pairwise *static λ* and *lytic λ* scores among four drugs are given as heatmaps. (top) Heatmaps correspond to best 4-order combinations against drug sensitive or resistant M. tuberculosis. The scores above heatmaps indicate the estimated 4-order interaction scores. (**a**) eta + fus + pre + van is the lowest ranked 4-order combination in our framework. (**b**) Since rif and van are similar drugs, we cannot use van against a rif-resistant Mtb strain. Therefore, the lowest ranked 4-order combination shown in (**a**) is not applicable. When rif and van are excluded, the lowest ranking 4-order combination is clz + fus + inh + sq1. (**c**) Similarly, since inh and eta are similar drugs, we cannot use eta against an inh-resistant Mtb strain. The best 4-order combination against inh-resistant strain is found as fus + pre + sq1 + van. (**d,e**) The best 4-order combinations against Mtb strains resistant to rif-inh or rif-inh-mox. (**f**) The best 4-order combination that includes current Mtb treatment. (bottom) Magenta and green frames show the 2-order combinations nominated for cycling. The two scores correspond to measured *static λ* and *lytic λ* scores for each pairwise combination. (**g**) Best 2-order combination cycling is pre + van and bdq + clz. (**h**–**k**) Best 2-order cycling options for Mtb strains resistant to indicated drugs. (**l**) When considered as two 2-order combinations, EIR + clz is split into clz + inh and eth + rif. For each resistance scenario and EIR + clz, the *static λ* and *lytic λ* scores in cycling is superior to 4-order combinations.

Above we searched best high-order combinations among combinations that include EIR. There are only 17 such 4-order combinations. However, there are 4845 4-order combinations among 20 drugs. To find the best 4-order combination among all 4,845 4-order combinations, we use a single additional exclusion criterion in R/ED that combination order = 4. The 4-order combination with the lowest meta-rank was eta + fus + pre + van, which has *static λ* = −0.49 and *lytic λ* = −0.42 (Fig. 5a). This is a vast improvement on EIR + clz, which had *static λ* and *lytic λ* of −0.16 and −0.25, respectively. This is due to several antagonistic interactions among members of EIR + clz (e.g. eth + inh, clz + eth, inh + rif), while there are no antagonistic interactions among eta + fus + pre + van. Importantly, this combination does not have similar drugs, as all such combinations are excluded from consideration.

To apply our model to regimens for MDR Mtb, we searched for the best 4-order combinations when the pathogen strain is resistant to one or more drugs. In the R/ED framework, this corresponds to adding two new exclusion criteria: (i) The combination must not have the drug to which the cell is resistant. For example, the combination against a rif-resistant pathogen should not include rif. (ii) The combination should not include any drug that is similar to the drug to which the cell is resistant. Since similar drugs are likely to have similar mechanisms of action, resistance to one drug may be accompanied by resistance to the similar drug. For example, the combination against a rif-resistant pathogen should not include van, which was similar to rif. Then, the best 4-order combination we identified (eta + fus + pre + van) is not applicable against rif-resistant Mtb since it includes van. The best 4-order combination for rif-resistant Mtb was clz + fus + inh + sq1, with *static λ* and *lytic λ* scores −0.37 and −0.27, respectively (Fig. 5b).

Similarly, a combination against inh-resistant should not have inh, eta or bdq. Since the best 4-way combination (eta + fus + pre + van) included eta, it is not applicable to inh-resistant Mtb. We find that a minor change in the best 4-order combination, replacing inh with sq1, results in the best 4-order combination against inh-resistant Mtb (Fig. 5c). When Mtb is resistant to both inh&rif, the 4-order combination should exclude inh and rif, as well as all inh similar (eta, bdq) and rif similar (van) drugs. clz + fus + mox + sq1 was the best 4-order combination against inh&rif resistant Mtb (Fig. 5d). For a strain that is resistant to rif&inh&mox, the best 4-order combination was fus + las + pre + sq1 (Fig. 5e). Therefore, R/ED can nominate best combinations from a given combination order under various drug resistance conditions. We contrast the 4-order combinations in Fig. 5a–e with the 4-order combination EIR + clz, shown in Fig. 5f. EIR + clz has inferior *static λ* and *lytic λ* compared to all other best combinations. This is expected, since it is the best among only 17 combinations, whereas 5a, 5b, 5c, 5d and 5e shows the best among 4845, 3060, 2380, 1365 and 1001 4-order combinations, respectively.

### Exploring best 2-order drug cycling options against drug-resistant *M. tuberculosis*

The analysis of 4-order combinations showed that even the best high-order combinations do not have strong static or lytic synergy. As synergy is rare and antagonism is common, this is to be expected. For each high-order combination, there are numerous pairwise antagonisms that contributes to an overall regression towards antagonism. In fact, the best 4-order combination described in Fig. 5a has only one static synergy and two lytic synergies, and is best since it avoids antagonistic combinations. When four drugs are mixed in a 4-order combination, both desired synergy and undesired antagonism will occur simultaneously. Therefore, we next considered using four drugs as cycles of 2-order combinations, where the same total dose of drugs is used, but these drugs are given at two different times. For example, if a treatment with a 4-order combination (a + b + c + d) involves using one dose of each drug twice, separated by one week; a 2-order cycling involves using a + b in two doses, followed by c + d in two doses after one week. In both cases, equal dose of each drug is used in treatment. However, 4-order synergies are weak and very rare, while pairwise synergies are strong and less rare. If a + b and c + d are synergistic, then cycling of a + b and c + d will have stronger synergy than a + b + c + d. In addition, since the treatment involves the same dose of drugs as the 4-order combination, we may regard the effect on resistance as equivalent. Since they offer an improvement in treatment, we next sought 2-order combination cycling options for different resistance scenarios.

To find best 2-order combination cycles, we found the pairwise combination with the best static and lytic rank and marked this pair as the first 2-order combination. We next found the pairwise combination with the next best rank that (i) does not have any common drug with first combination, (ii) does not have any drugs similar to drugs in the first combination. We marked this pair as the second 2-order combination. Figure 5g shows the best 2-order combination cycle: pre + van and bdq + clz. Each heatmap shows the pairwise *static λ* and *lytic λ* scores among four drugs in consideration. Two 2-order combinations to be used in cycling are framed in magenta and green. Above each heat map, *static λ* and *lytic λ* scores of the 2-order combinations are given. It is apparent that when considered as a 4-order combination, pre + van + bdq + clz has a high rank due to strong static and lytic antagonism between clz and pre. However, when these four drugs are considered as two 2-order combinations, clz and pre are never combined and clz + pre antagonism does not occur. The best 2-order cycling had much better *static* and *lytic λ* scores than the best 4-order combination, improving with a score of almost 1, corresponding to 50% dose reduction to achieve the same effect.

Similar to the considerations in 4-order combinations, resistance backgrounds constrict the sample space where 2-order cycling options can be searched. Above, we found that pre + van and bdq + clz is the best 2-order combination cycling. Since van and rif are similar drugs, this solution is not applicable when Mtb is resistant to rif. We find that replacing van with eta, hence a cycling of pre + eta and bdq + clz, is the best 2-order combination cycling against rif-resistant Mtb. Similarly, the best solution is not applicable to inh-resistant Mtb, since it includes bdq, which is similar to inh. We find that replacing bdq with fus, hence a cycling of pre + van and fus + clz, is the best 2-order cycling in this case (Fig. 5c). For multidrug-resistant strains (Fig. 5j,k) clz + fus and las + pre cycling were the best option. Finally, we considered the improvement on EIR + clz if it was used as a 2-order combination cycle. We find that cycling of clz + inh and eth + rif is vastly superior to the 4-order combination in regard to static and lytic synergy scores.

## Discussion

High-order combinations of drugs with non-overlapping resistance mechanisms are integral to the treatment of complex diseases. However, it has been recently shown that the standard drug cocktail against Mtb is not synergistic. A synergistic high-order combination of drugs can provide an attractive alternate means of Mtb treatment. Here, developed R/ED, an expandable and transferrable model to find high-order combinations with best synergy properties and with non-overlapping resistance. Given specific disease contexts, even additive combinations might be useful for treatment. R/ED provides a ranking of all possible combinations among drugs, allowing the selection of combinations with desired properties.

While R/ED currently uses bacteriostatic and lytic synergies for ranking, new phenotypes can be integrated to the meta-rank for each combination. When drugs are combined in a patient, the combination may be toxic, in phenomena known as adverse drug interactions. In the current study, all observations were done on Mtb cells *in vitro* and no data on toxic effects were used. A future study may expand R/ED by incorporating toxicity information. Another R/ED expansion may use different weights for different desired outcomes. For example, a toxic effect is definitely not wanted, but a treatment would still be suitable if synergy is weak. In this case, the adverse drug interaction rank would have a larger weight while effecting the meta-rank in comparison with the weight of synergy. Similarly, new exclusion criteria can be appended to R/ED. For example, drugs may have different pharmacokinetic properties and reach peak concentration in the target compartment in different times. Such a scenario would prevent the realization of the synergy that was observed *in vitro*: To interact, drugs must have similar bioavailability profiles. A new criterion that excludes drugs pairs with different pharmacokinetic properties may expand the R/ED framework and provide a more realistic representation of treatment effects. This is especially relevant for combinations involving many drugs. For example, we have nominated one 14-order combination with better synergy than canonical tuberculosis treatment. However, delivering all drugs to the target compartment simultaneously is more difficult with increasing number of drugs. This observation exemplifies the fact that while our framework provides candidate high-order combinations for treatment, these combinations will require further development stages before being clinically relevant. Analysis of possible adverse interactions among the constituents of the combinations will be one of these stages, addressing the safety and toxicity of the high-order combination.

R/ED framework is immediately transferrable to other model systems and diseases. While our study focused on Mtb, the experiment and the analysis pipelines are agnostic to the model system. R/ED could be directly applied to the discovery of high-order combinations against other pathogens and tumors. Our study exemplifies that acquiring a data set for R/ED is within reach and provides a template for experimental design in other model systems. For any given cancer or pathogen, a list of 20 drugs often covers all different drugs that can be used. Here we used 40 384-well plates to measure all pairwise combinations among 20 drugs. With such a low cost, we hope similar screens will be conducted for finding best high-order combinations against tumors and other pathogens.

In this study, we conducted the largest experimental screen for pairwise bacteriostatic interactions in Mtb. We also reanalyzed the screening data to generate lytic interaction scores for the measured antibiotic pairs. It should be noted that lytic effect is not equivalent to cidal effect. Cells can die without lysis. Moreover, a slowly killing antibiotic will cause some OD increase prior to growth inhibition and toxicity. The consensus approach for cidality measurement is the count of colony forming units. This measure directly reflects the number of dead cells after a treatment. However, given the extremely long doubling time of Mtb, such measurements have not yet been performed in the scale we used in our study. When Mtb cidal interaction scores are available, they can be included in our framework to improve treatment predictions.

We have argued that synergistic drug interactions can be best exploited using a sequential drug regime. However, it has been shown that antibiotics can induce long lasting physiological changes, in phenomena known as cellular hysteresis^33^. Such changes may influence the effect of another drug and the hysteresis landscape includes negative, positive, and neutral effects with strong directionality. Hence, cellular hysteresis emerges as an important parameter while designing sequential drug regimes. The effect of cellular hysteresis is equally important while designing sequential combination regimes. A better understanding of this phenomenon will lead to the improvement of the best treatment options we have suggested in our analysis. This points out to the importance of generating large-scale cellular hysteresis data, which can be utilized in computational models.

While high-order combinations are useful for slowing down resistance evolution, they have weaker synergy. This observation may provide the following rule of thumb when considering the use of high-order combinations for treatment: If toxicity or efficacy is the major concern, then synergy should be prioritized. If drugs with narrow therapeutic windows are to be used, then the total dose in a synergistic combination may be lowered, achieving the same efficacy with lower dose. If drugs have large therapeutic windows, keeping the dose of each drug constant will increase the efficacy of a synergistic combination. If resistance evolution is the major concern, then high-order combinations should be prioritized. For example, the treatment of RNA viruses with very fast evolution rates may benefit from ultra-high order combinations, even if the combination is additive.

## Methods

### Experimental drug interaction screen

Pairwise drug interactions were measured using the diagonal method^21,34^. All drugs were dissolved in DMSO and stored at −20 °C. Using a digital drug dispenser (D300e Digital Dispenser, HP), we printed linearly increasing doses of individual drugs or 2-order combinations in 384-well plates. Each drug or combination was printed along one column of the plate, excepting the top and bottom rows to minimize edge effects, corresponding to 14 wells per drug or combination. Our experimental design included each individual drug in a combination on the same plate with the combination to account for plate effects. The top concentration at the 14^th^ well of each individual drug is given in Table 1. Pairwise combinations receive half of this top concentration for each drug. Mtb cells are grown on these drugs and combinations for five days and the optical density reached by the growing Mtb population is recorded by a plate reader. 36 of these interaction scores were previously reported while developing the experimental design used in our study.

### Data analysis and R/ED framework

We rearranged our interaction screen data for a more streamlined analysis. Using the raw data, we produced one data file per replicate pairwise interaction experiment, 380 interaction files in total (Additional File 2). Each file contains three columns, corresponding to optical density readings in two individual drugs and the combination; and 14 rows, corresponding to increasing doses of each drug or combination. For each interaction experiment, we found the well in each column that inhibits growth by 70% and defined this well as the IC70 (70% inhibitory concentration). Using the IC70 for each single drug, we calculated an expected IC70 for the combination column. The ratio of the observed IC70 by the expected IC70 gives the FIC (Fractional Inhibitory Concentration), a Loewe additivity model based drug interaction score. This score is 1 for additive pairs; (0,1) or >1 for synergistic or antagonistic pairs, respectively. In this study, we used *static λ* = log2(FIC) as our drug interaction measure. This score is 0, <0 or >0 for additive, synergistic or antagonistic pairs, respectively. For a pair a + b, a *static λ* of −1 means that 0.25 dose of a + 0.25 dose of b (half total dose) has the same effect with a full dose of a or a full dose of b. We used the mean of two replicates as the *static λ* for each pair. We assigned *static λ* scores to high-order combinations by computing the mean of all 2-order *λ* among the constituents of the combination. Throughout the study, we name combinations by alphabetical ordering of their content.

For lytic interaction measurement of a pair, we found the well in each column that has an optical density of 0.12, which corresponds to the lysis of 50% of the initial Mtb population. We defined this well as the LC50 (50% lytic concentration). In cases where 50% lysis was not observed, we used the top dose as LC50. Using the LC50 for each single drug, we calculated an expected LC50 for the combination column. Similar to the FIC calculation described above, ratio of observed LC50 by expected LC50 provides a lytic synergy measure, which we will call FLC (Fractional Lytic Concentration). We used *lytic λ* = log2(FLC) as our lytic drug interaction measure, which is interpreted the same way with FIC. When neither the individual drugs nor the combination had LC50, we were unable to assign a *lytic λ* score to a pair (112 pairs). As final lytic interaction scores, we used the mean of two replicates when available. We assigned lytic scores to high-order combinations by computing the mean of all 2-order *lytic λ* scores among the constituents of the combination. We used the mean of all known 2-order *lytic λ* (−0.07) as an approximation for the 2-order combinations for which *lytic λ* was not measured.

## Data Availability

The datasets supporting the conclusions of this article are available in the figshare repository https://figshare.com/s/1e4c388e2727e90e3cc6. Additional file 1 contains drug ingredients, static *λ* scores, lytic *λ* scores and number of drugs for each of the 220 combinations studied. Additional file 2 contains all raw data collected during this study. 380 files correspond to two replicates for 190 drug interaction experiments. Each file contains three columns, corresponding to growth measurements in two single drugs and their combination.
